# On Comparison of Series and Numerical Solutions for Flow of Eyring-Powell Fluid with Newtonian Heating And Internal Heat Generation/Absorption

**DOI:** 10.1371/journal.pone.0129613

**Published:** 2015-09-24

**Authors:** Tasawar Hayat, Shafqat Ali, Muhammad Asif Farooq, Ahmad Alsaedi

**Affiliations:** 1 Department of Mathematics, Quaid-i-Azam University, 45320, Islamabad, 44000, Pakistan; 2 Faculty of Engineering Sciences, Ghulam Ishaq Khan Institute of Engineering, Sciences & Technology, 23460, Topi, 44000, Pakistan; 3 Centre for Advanced Mathematics and Physics (CAMP), National University of Sciences and Technology (NUST), Sector H-12, Islamabad, 44000, Pakistan; 4 Department of Mathematics, Faculty of Science, King Abdulaziz University, P. O. Box. 80257, Jeddah, 21589, Saudi Arabia; Tianjin University, CHINA

## Abstract

In this paper, we have investigated the combined effects of Newtonian heating and internal heat generation/absorption in the two-dimensional flow of Eyring-Powell fluid over a stretching surface. The governing non-linear analysis of partial differential equations is reduced into the ordinary differential equations using similarity transformations. The resulting problems are computed for both series and numerical solutions. Series solution is constructed using homotopy analysis method (HAM) whereas numerical solution is presented by two different techniques namely shooting method and *bvp*4*c*. A comparison of homotopy solution with numerical solution is also tabulated. Both solutions are found in an excellent agreement. Dimensionless velocity and temperature profiles are plotted and discussed for various emerging physical parameters.

## Introduction

Flow analysis of non-Newtonian fluid has received growing interest in the past few decades. These types of fluids occur in engineering, biology and industry etc. Some common examples of non-Newtonian fluids are certain paints, blood at low shear rate, ketchup, shampoo, toothpaste, salvia, synovial fluids, sewage sludge, foams and emulsions etc. Due to their occurrence in biological and industrial processes, the research on non-Newtonian fluid has been presented through different aspects. It is now well established fact that the flows of all the non-Newtonian fluids cannot be examined by one constitutive relationship between shear rate and stress. This happens in view of the diverse characteristics of non-Newtonian fluids. Hence several constitutive equations have been proposed subject to classification of non-Newtonian fluids through differential, rate and integral type. Also, the governing equations of non-Newtonian fluids are more complex and non-linear than the Navier-Stokes equations. Infact, the rheological parameters in the constitutive equations make the governing problems more tedious[1–5].

The flows of non-Newtonian fluids with heat transfer are also of practical interest in industrial applications including multiphase mixtures, biological fluids, food products, agriculture and dairy wastes and natural products. Interests of recent researchers in such flows has grown regarding control of the quality of the final product in various manufacturing and processing industries such as hot rolling, continuous casting, wire drawing, glass fiber production, aerodynamic extrusion of polymer sheets and paper production. In all these processes, the rates of cooling and stretching have a vital role. Therefore, several investigators even in recent times are engaged for the boundary layer flows generated by a stretching surface. For instance, Layek et al. [6] presented the boundary layer stagnation point flow towards a permeable stretching surface with heat and mass transfer. Nadeem et al. [7] constructed the analytic solution for stagnation point flow of a stretching sheet. Bhattacharyya and Layek [8] addressed the influence of suction/ blowing on the two-dimensional stagnation point flow. Yacob et al. [9] explored the melting heat transfer analysis in stagnation point flow of micropolar fluid bounded by a stretching/shrinking surface. The slip flow and heat transfer over a permeable surface in a porous medium is studied by Bhattacharyya et al. [10] Influence of thermal radiation on the boundary layer flow induced by a porous moving surface is discussed by Mukhopadhyay et al. [11] Bhattacharyya and Layek [12] analyzed the MHD flow generated by a permeable stretching sheet with chemically reactive solute distribution. Boundary layer flow of power law fluid bounded by a stretching through Lie group approach is analyzed by Jalil and Asghar [13] Ahmad and Asghar [14] studied the MHD flow of second grade fluid over a stretching surface with arbitrary velocities. Hayat et al. [15] explored simultaneous effects of heat and mass transfer in time-dependent flow by a stretching surface. MHD flow of chemical reactive UCM fluid past a permeable surface is presented by Vajravelu et al. [16]

It is noted from the above mentioned studies and many others that heat transfer characteristic in boundary layer flow is studied much either through prescribed heat flux or prescribed surface temperature. No reasonable attention is given to the flows subject to Newtonian heating from the surface. Few studies in this direction have been reported. For instance, Merkin [17] studied the natural convection boundary layer flow on a vertical surface with Newtonian heating. Salleh et al. [18] examined the boundary layer flow and heat transfer over a stretching sheet with Newtonian heating. Lesnic et al. [19] analyzed the free convection boundary layer flow along a vertical surface in a porous medium with Newtonian heating. The boundary layer flow of forced convection at a forward stagnation point with Newtonian heating is presented by Salleh et al. [20] Chaudhary and Jain [21] also constructed an exact solution to the unsteady free convection boundary-layer flow past an impulsively started vertical surface with Newtonian heating. Stability of thermal convection of an Oldroyd-B fluid in a porous medium with Newtonian heating is studied by Niu et al. [22] Some recent development in the study of multiphase flow is given by Zhong et al. [23–25].

The objective of present communication is to explore the effect of Newtonian heating in the boundary layer flow of Eyring-Powell fluid [26] Consideration of this fluid has importance in the sense that it correctly reduces to viscous case at low and high shear rates. Further it is deduced from kinetic theory of liquids rather than the empirical relation. The flow in this attempt is caused by a stretching surface. Analysis has been carried out in the presence of heat generation/absorption. This concept is of vital importance in applications such as those involving heat removals from nuclear fuel debris, underground disposal of radioactive waste material, storage of food stuffs and exothermic chemical reactions and dissociating fluids in packed-bed reactors. The rest of the paper is organized in the following fashion. Next section consists of problem formulation. In section three, Series solutions have been obtained by homotopy Analysis method (HAM) [27–32] whereas the numerical solution is obtained by *bvp*4*c* and shooting method. In section four, comparison of HAM solution and numerical results are tabulated. Also the effects of various physical parameters are ploted and analyzed.

## Formulation of the Problems

We consider the steady boundary layer flow of an incompressible Eyring-Powell fluid over a stretching surface at *y* = 0. We are interested to model the analysis in the presence of heat source/sink. The stretching sheet possesses the effects of Newtonian heating. The boundary layer flow in the present situation is governed by the following expressions.

$$\frac{\partial u}{\partial x}+\frac{\partial v}{\partial y}=0,$$ (1)

u∂u∂x+v∂u∂y=(ν+1ρβ1C)∂2u∂y2−12ρβ1C3(∂u∂y)2∂2u∂y2 (2)

$$u\frac{\partial T}{\partial x}+v\frac{\partial T}{\partial y}=\alpha_{m}\frac{\partial^{2}T}{\partial y^{2}}+\frac{Q_{0}}{\rho C_{p}}(T-T_{\infty }),$$ (3)

with the following prescribed conditions

$$\begin{array}{l} \;u=u_{w}(x)=ax,\;v=0,\;\frac{\partial T}{\partial y}=-h_{s}T\;\text{at}\;y=0,\\ u\to 0,\;T\to T_{\infty }\;\text{as}\;y\to \infty . \end{array}$$ (4)

In above expressions *u* and *v* are the velocity components along the *x* and *y* − directions respectively, *β*_1_ and *C* are the material parameters, *v* is the kinematic viscosity, *ρ* the fluid density, *T* the temperature of fluid, *T*_*∞*_ is the temperature of fluid for away from the surface, *Q*_0_ is the dimensional heat generation/absorption coefficients, *C*_*p*_ the specific heat at constant pressure and *α*_*m*_ is the thermal diffusivity of ordinary fluid.

We proceed for solutions through stream function satisfying

$$u=\frac{\partial \psi }{\partial y}\;,\;v=-\frac{\partial \psi }{\partial x}\;,$$ (5)

and

$$\psi =x\sqrt{c\;\nu }f(\eta ),\;\theta (\eta )=\frac{T-T_{\infty }}{T_{\infty }},\;\eta =\sqrt{\frac{c}{\nu }}y.$$ (6)

Now Eq (1) is clearly satisfied and Eqs (2–6) give

(1+ε)f‴+ff″−f′2−εδf′′2f‴=0, (7)

θ″+Prfθ′+Prλθ=0, (8)

$$\begin{array}{l} f\prime (\eta )=1,\;f(\eta )=0,\;\theta \prime (\eta )=-\gamma (1+\theta (\eta ))\;\text{at}\;\eta =0,\\ f\prime (\eta )=0,\;\theta (\eta )=0\;\text{as}\;\eta \to \infty , \end{array}$$ (9)

where *ε* and *δ* are the material fluid parameters, *λ* is the heat source/sink parameter, Pr the Prandtl number and *γ* the conjugate parameter for Newtonian heating. These dimensionless parameters are defined as

$$\varepsilon =\frac{1}{\mu \beta_{1}C},\;\delta =\frac{a^{3}x^{2}}{2\nu c^{2}}\;\text{Pr}=\frac{\nu }{\alpha_{m}},\;\gamma =h_{s}\sqrt{\frac{\nu }{a}},\;\lambda =\frac{Q_{0}}{a\rho c_{p}},$$ (10)

The local Nusselt number *Nu*_*x*_ and skin friction coefficient *C*_*f*_ are defined as

$$Nu_{x}=\frac{xq_{w}}{k(T-T_{\infty })},\;C_{f}=\frac{\tau_{w}}{\rho U_{w}^{2}},$$ (11)

in which the heat flux *q*_*w*_ is defined by the following relation

$$q_{w}=-k{\left(\frac{\partial T}{\partial y}\right)}_{y=0,}$$ (12)

with *k* being the thermal conductivity. In dimensionless form, the quantities in Eq (11) become

$${\left(R_{e_{x}}\right)}^{-1/2}Nu_{x}=\gamma \left(1+\frac{1}{\theta (0)}\right),\;C_{f}/{\text{Re}}_{x}^{-1/2}=\left(1+\varepsilon \right)f\prime \prime \left(0\right)-\frac{\varepsilon }{3}\delta f^{\prime \prime 3}\left(0\right),$$ (13)

where $R_{e_{x}}=(ax^{2}/\nu )$ is the local Reynolds number.

## Analytical and Numerical Solutions

Here analytical solution is obtained by homotopy analysis method and numerical solution is presented by MATLAB fnite difference built-in-function *bvp*4*c*.

### Series solution

**Fig 1:**
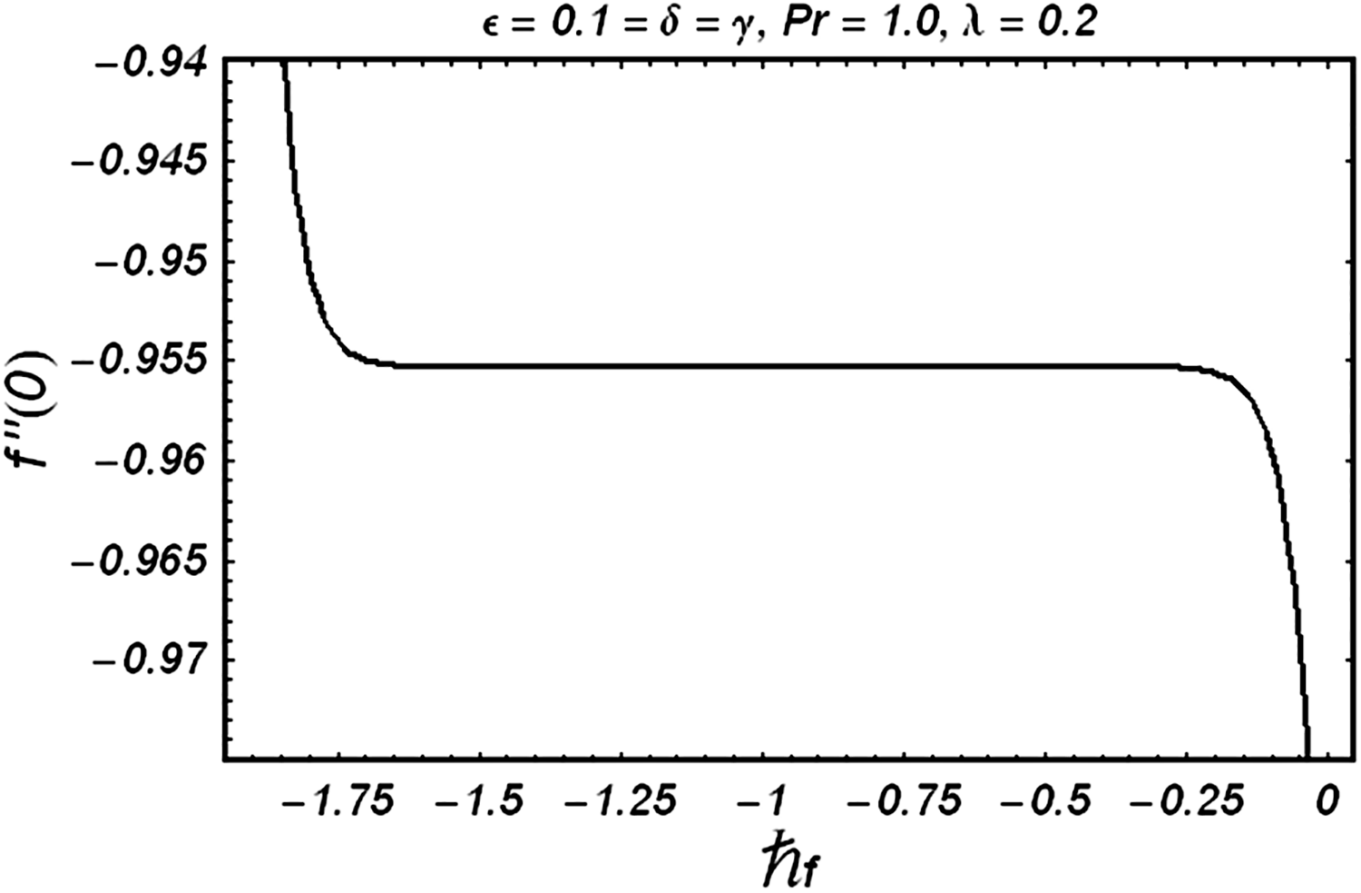
ℏ_*f*_ curve for velocity.

**Fig 2:**
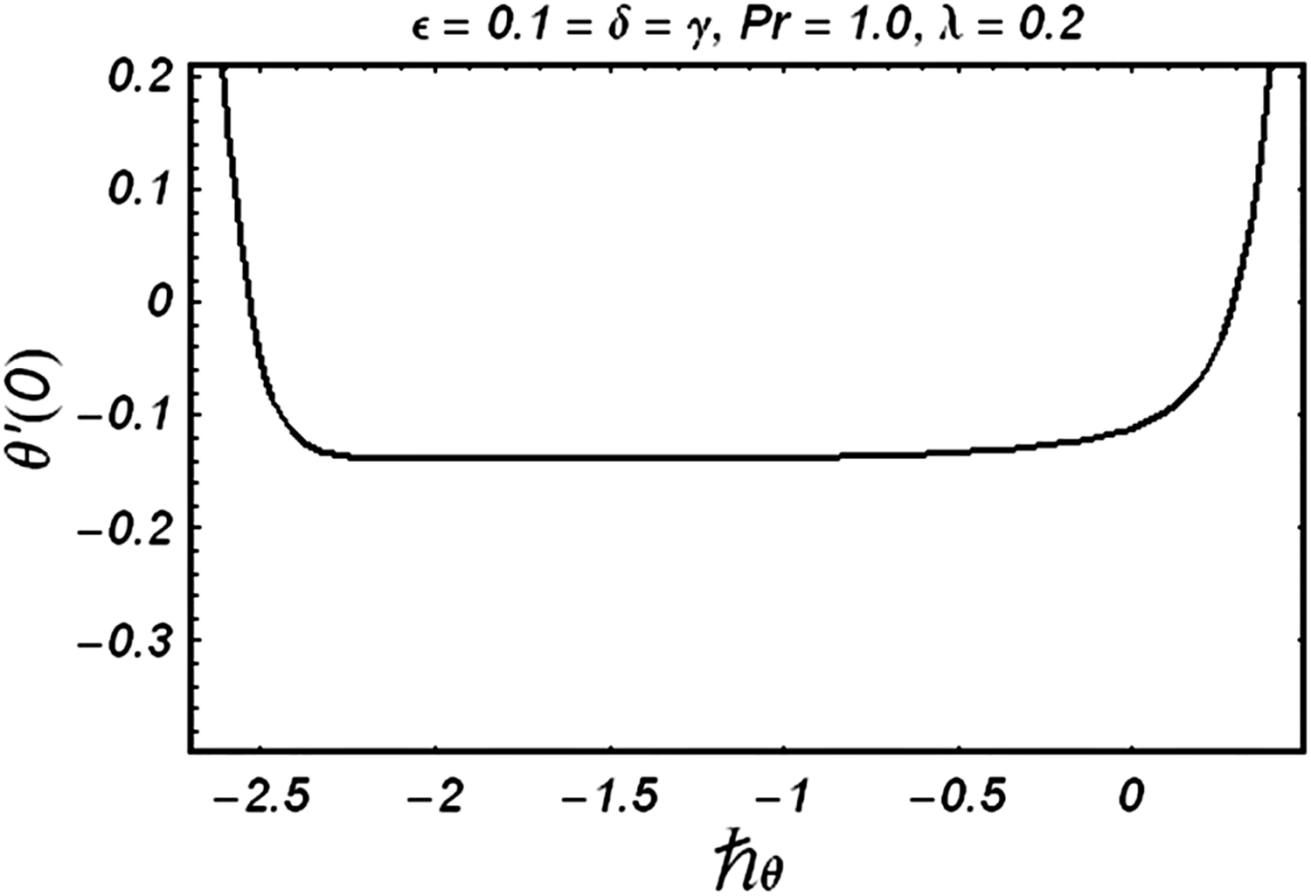
ℏ_*θ*_ curve for temperature.

In this section, Eqs (7) and (8) subject to the boundary conditions (9) are solved using homotopy analysis method. We choose auxiliary parameters ℏ_*f*_ and ℏ_*θ*_ for the functions *ƒ* and *g* respectively. The convergence of the obtained series solutions strictly depends upon these parameters. In order to obtain the permissible values of auxiliary parameters, we have plotted ℏ -curves in the Figs 1 and 2 for *ε* = 0.1 = *δ* = *γ*, *λ* = 0.2 and Pr = 1.0. (Fig 1) depicts that the range for acceptable value of ℏ_*f*_ is from −1.6 to –0.25. Fig 2 shows that the appropriate range for ℏ_*θ*_ is from –2.4 to –0.8.

Table 1 shows that 15^*th*^ order of approximation is sufficient for the convergence of series solution of velocity upto six decimal places whereas the solution for temperature converges at 20^*th*^ order of approximation.

**Table 1:** Convergence of the HAM solutions for different order of approximation when *ε* = 0.1 = *δ* = *γ*, Pr = 1.0, *λ* = 0.2.

Order of Approximation	-f”(0)	-θ′(0)
5	0.955130	0.129113
10	0.955989	0.134368
15	0.956018	0.138456
20	0.956018	0.139597
30	0.956018	0.139597
40	0.956018	0.139597

### Numerical solution

In this subsection, we have solved Eqs (7) and (8) numerically subject to BCs (9). Here, we have used two different numerical techniques, the higher order MATLAB finite difference built-in-function *bvp*4*c* and shooting method. The first step for using *bvp*4*c* in MATLAB is to transform Eqs (7) and (8) into a system of first order ODEs.

$$\begin{array}{l} f_{1}^{{}^{\prime }}=f_{2},\;f_{2}^{{}^{\prime }}=f_{3},\;f_{3}^{{}^{\prime }}=\frac{1}{1+\varepsilon -\varepsilon \delta }(-f_{1}f_{3}+f_{2}^{2}),\\ f_{4}^{{}^{\prime }}=f_{5},\;f_{5}^{{}^{\prime }}=-\text{Pr}\;f_{1}f_{5}-\text{Pr}\;\lambda f_{4}. \end{array}$$ (14)

where we have introduced (*f*_1_, *f*_2_, *f*_3_, *f*_4_, *f*_5_) = (*f*, *f*′, *f*″, *θ*, *θ*′). The BCs (9) are also written in a boundary value residual form as per requirement of bvp4c:

f02−1=0,f01=0,f05+γ(1+f04)=0,f∞2=0,f∞4=0. (15)

In above equation, *f*0 and *f∞* represents the left and right boundary points.

The MATLAB built-in *bvpinit* is used for the initial mesh and initial guess in BVP consisting of Eqs (7) and (8). The general form of bvpinit is written in MATLAB as:

sol=bvpinit(initialmesh,initialguess) (16)

The Eqs (14) and (15) are called in with using function handles and solution is added in the argument of *bvp*4*c* as follows

solution=bvp4c(@bvp,@bc,sol) (17)

The final form of the solution obtained with *bvp*4*c* in Eq (17) is in structure class of MATLAB. The grid points in *η* − *direction* and solution is extracted with *sol*.*x* and *sol*.*y*, respectively. The *sol*.*y* contains the following solution (*f*, *f*′, *f*″, *θ*, *θ*′). For detail about *bvp*4*c* consult reference [33].

For shooting method we implemented Newton-Raphson method to find the targets and adaptive Runge-Kutta method is chosen for the time integration in MATLAB.

## Comparison and Discussion

**Fig 3:**
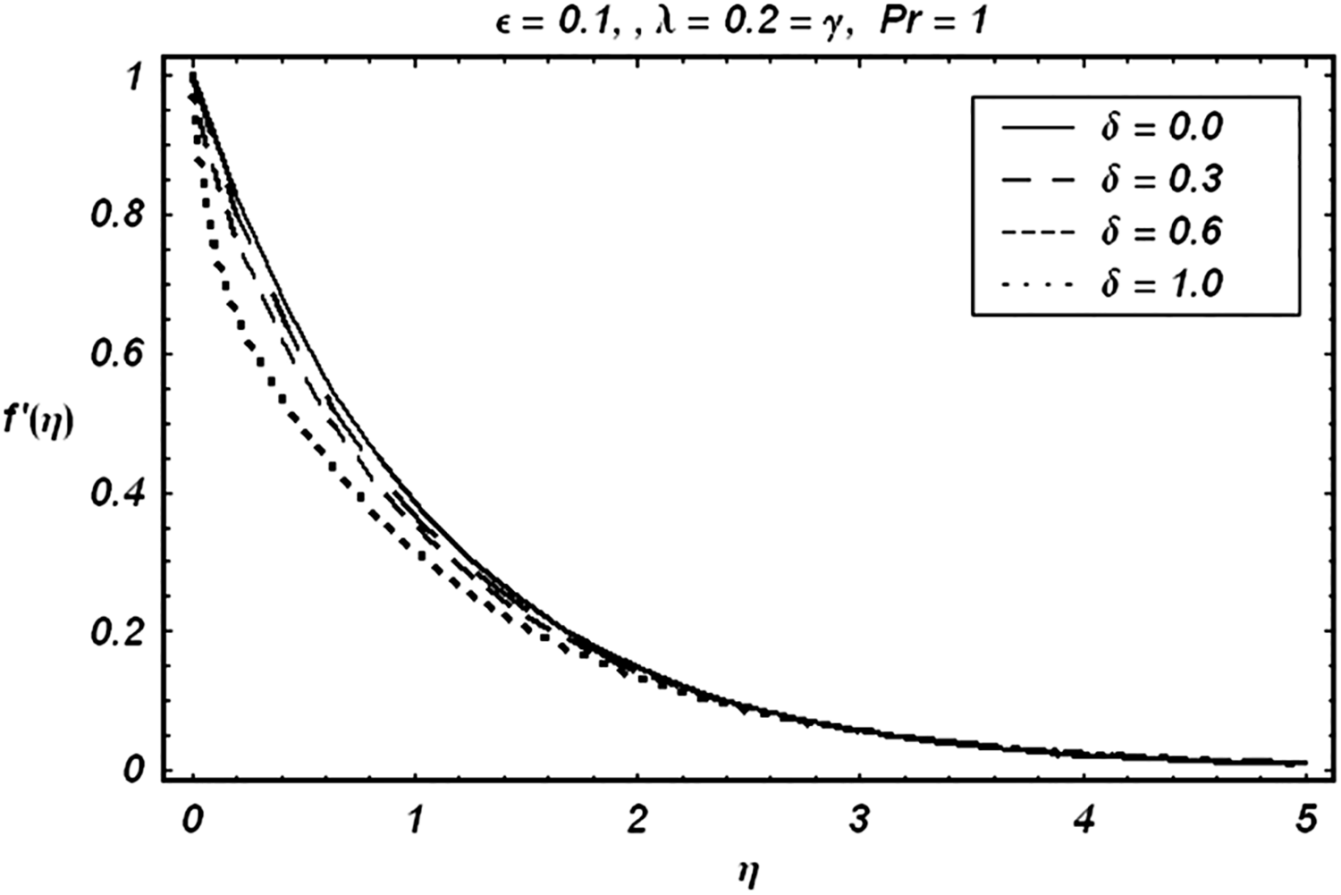
Variation of δ on f′.

**Fig 4:**
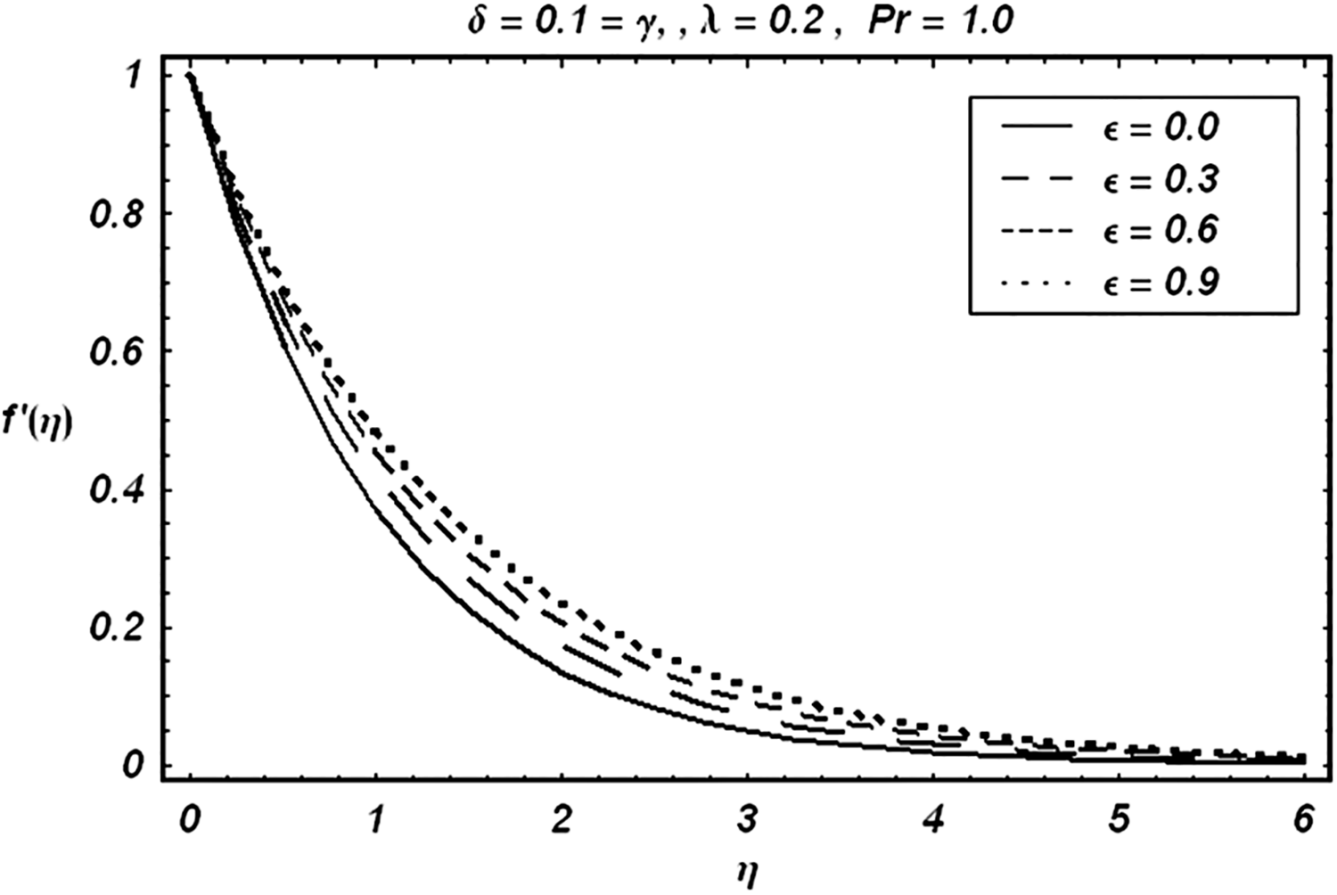
Variation of ε on f′.

**Fig 5:**
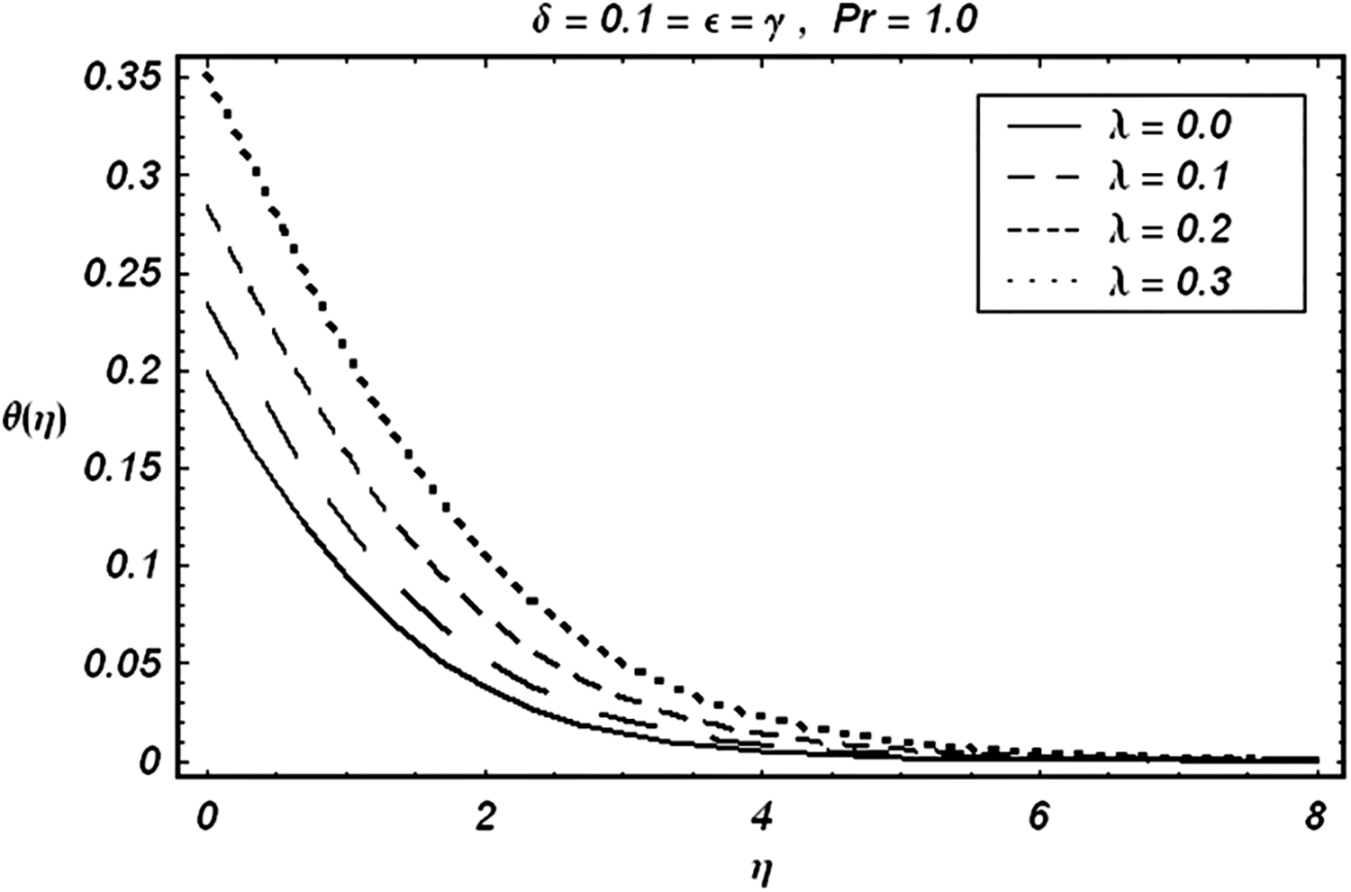
Variation of (λ>0) on θ.

**Fig 6:**
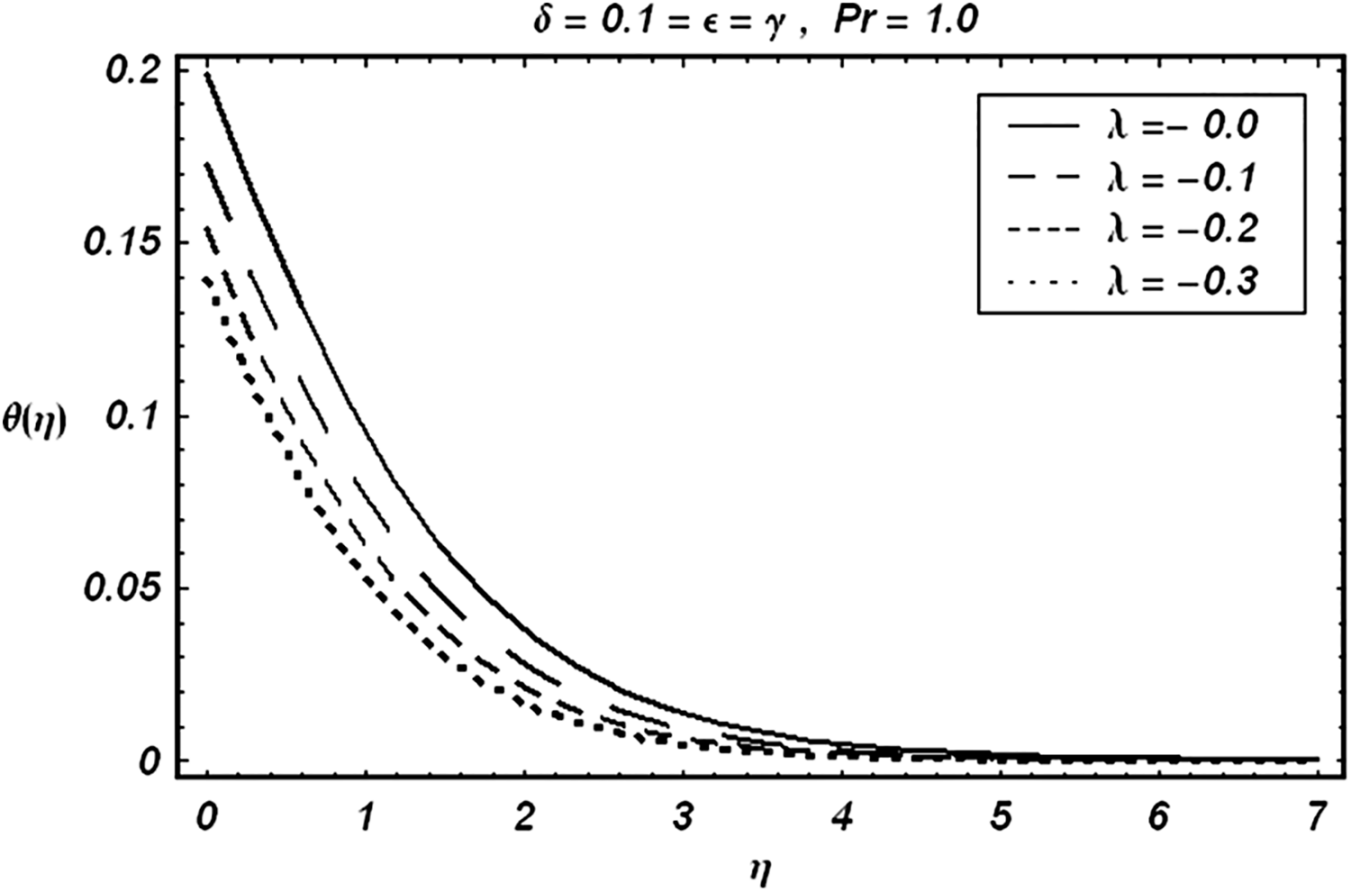
Variation of (λ<0) on θ.

**Fig 7:**
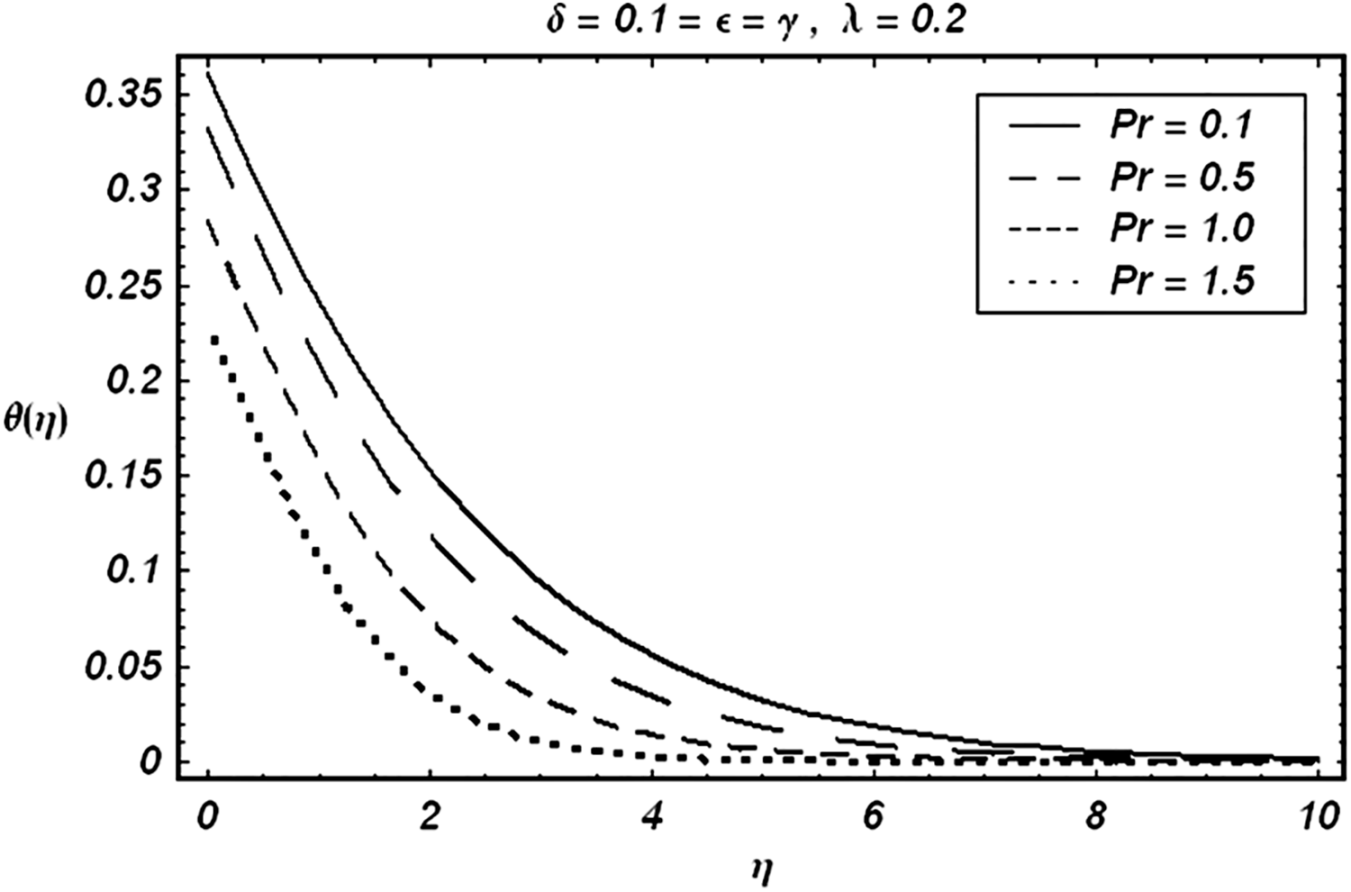
Variation of Pr on θ.

**Fig 8:**
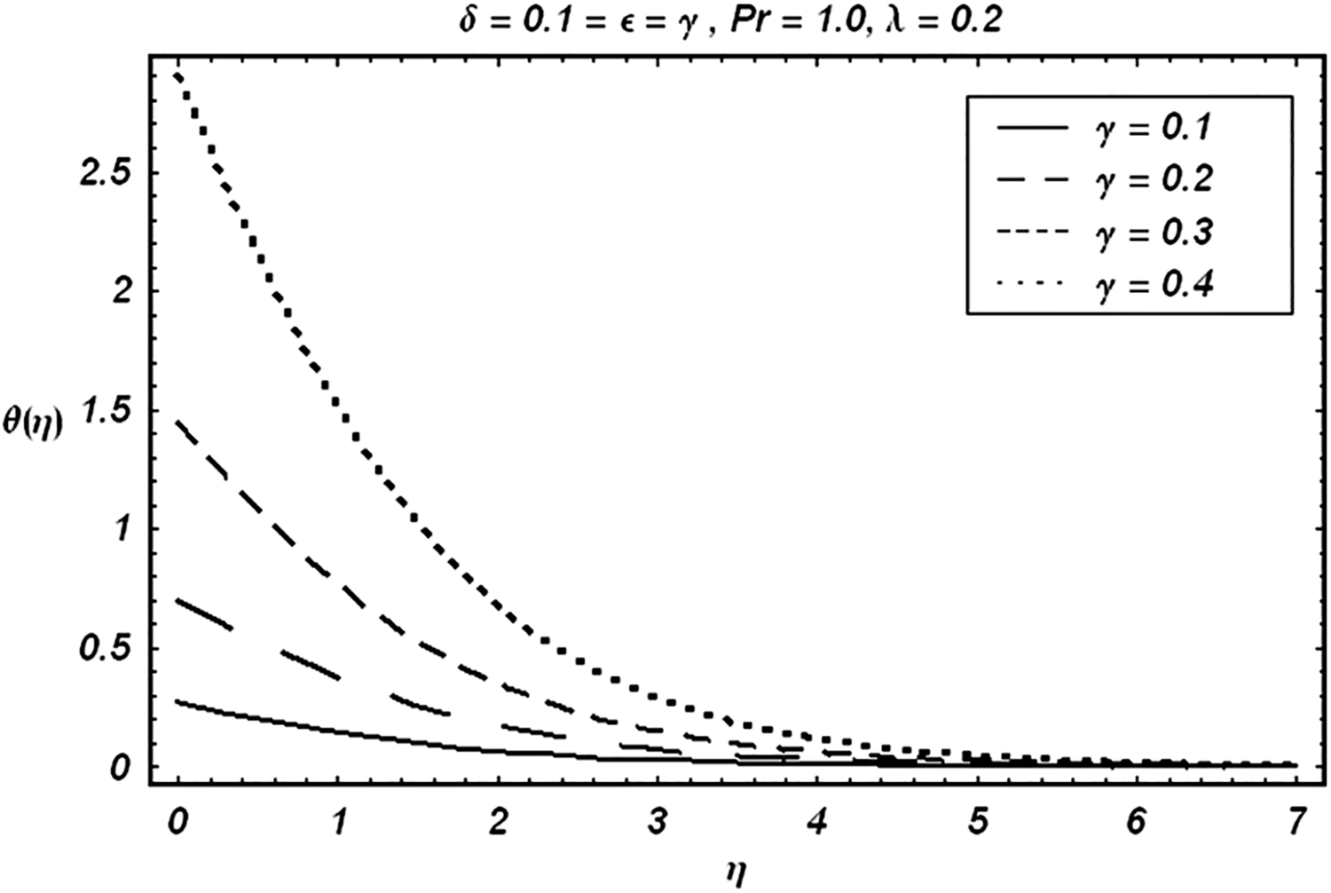
Variation of γ on θ.

**Fig 9:**
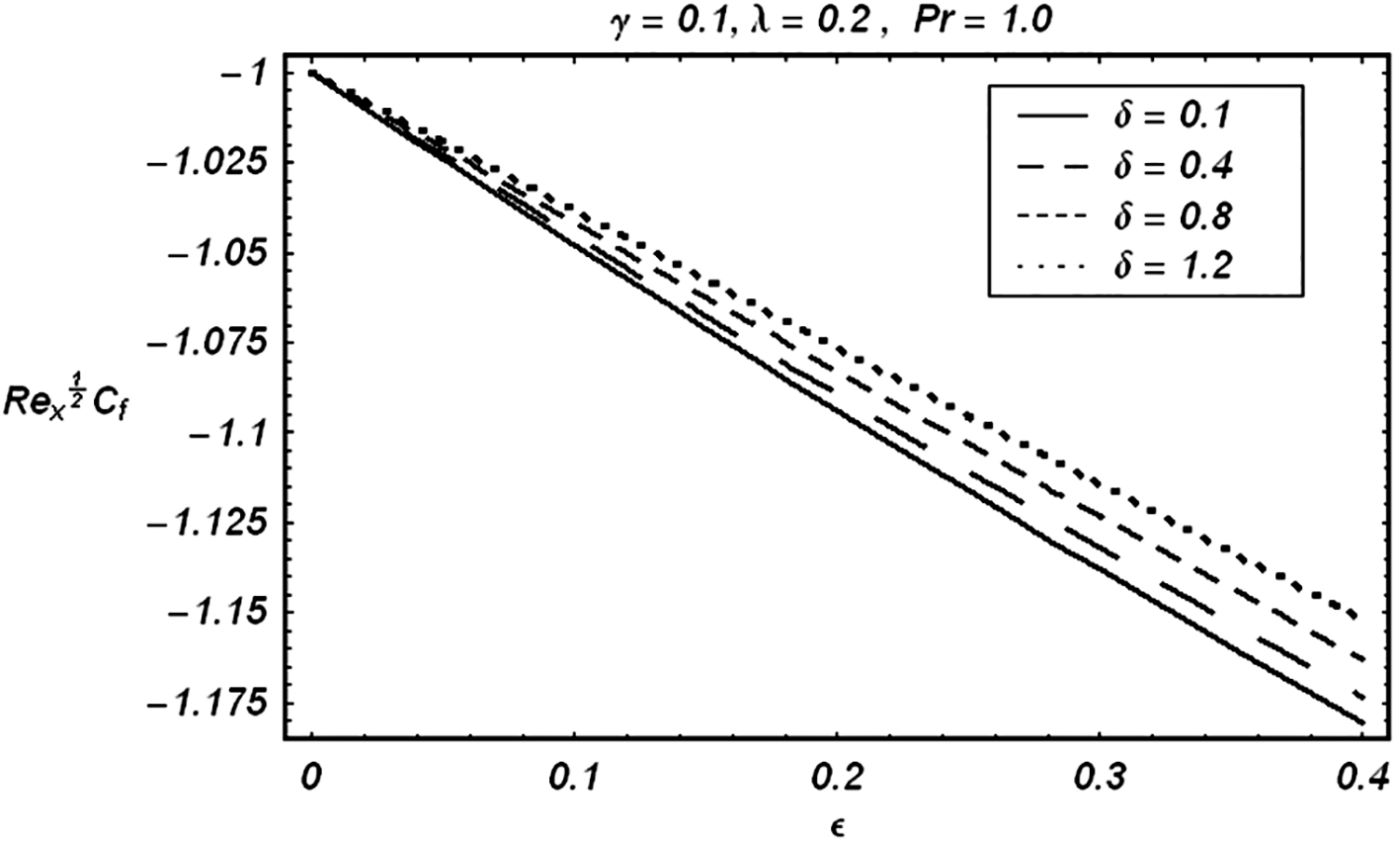
Effect of δ on skin friction.

**Fig 10:**
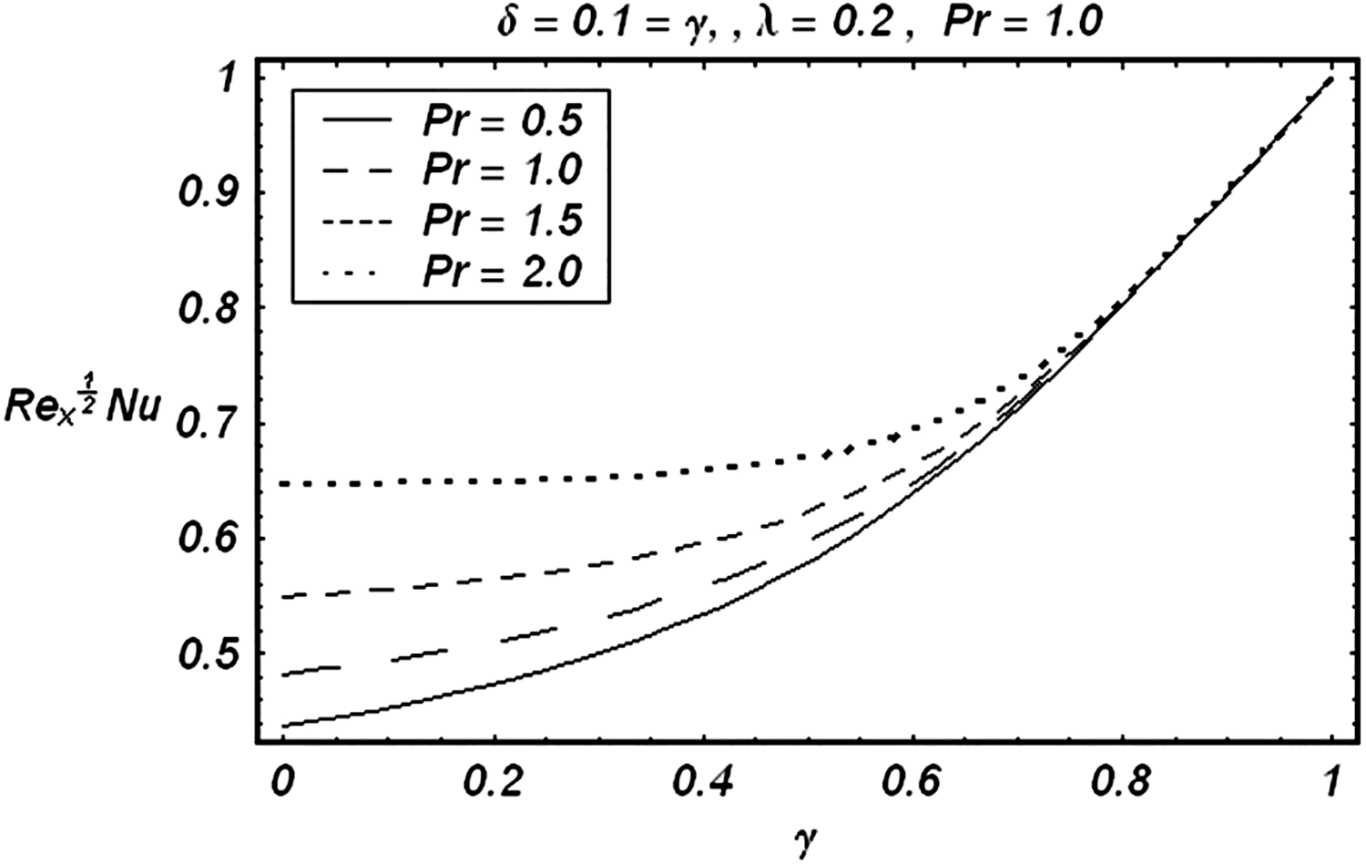
Effect of Pr on local Nusselt number.

**Fig 11:**
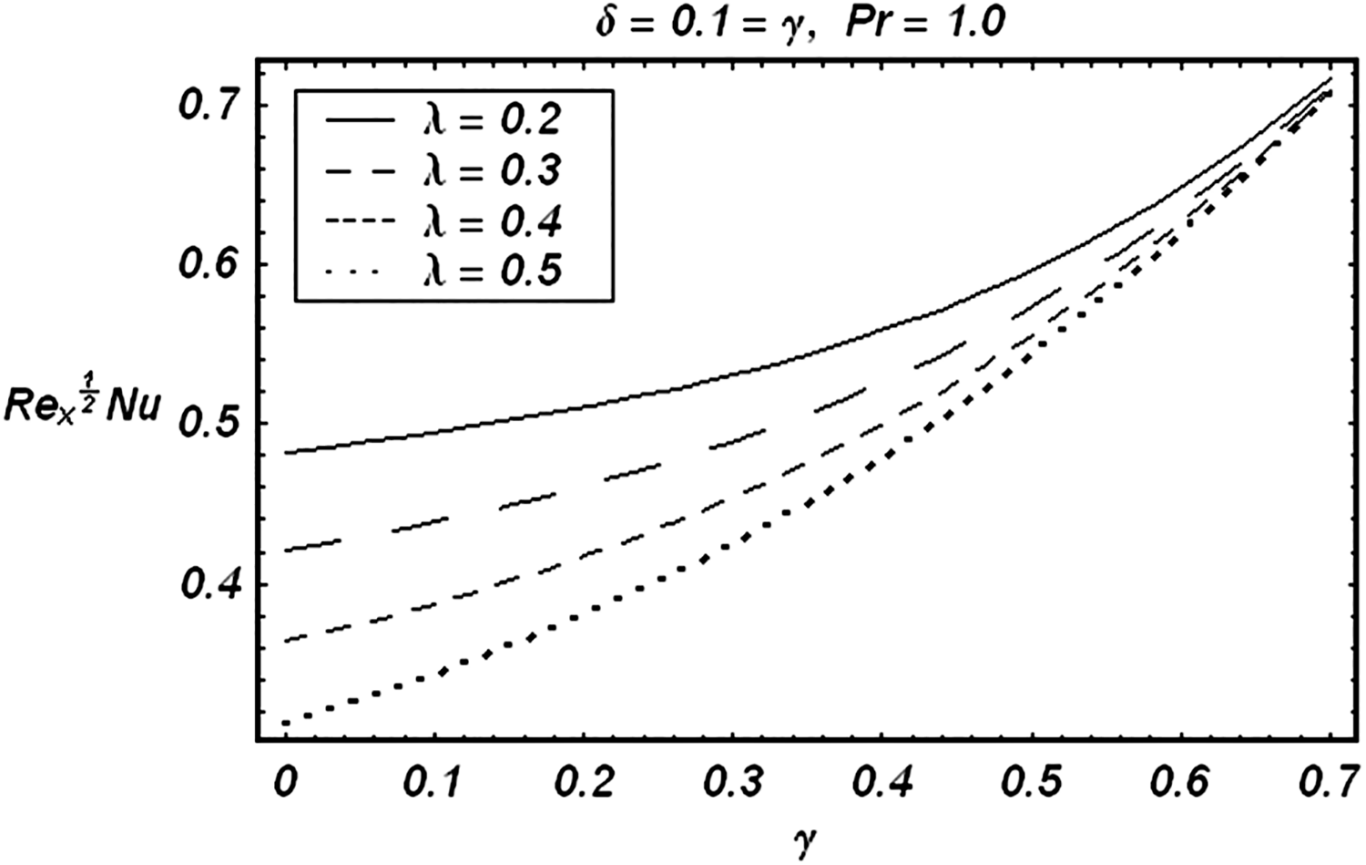
Effect of (λ>0) on local Nusselt number.

**Fig 12:**
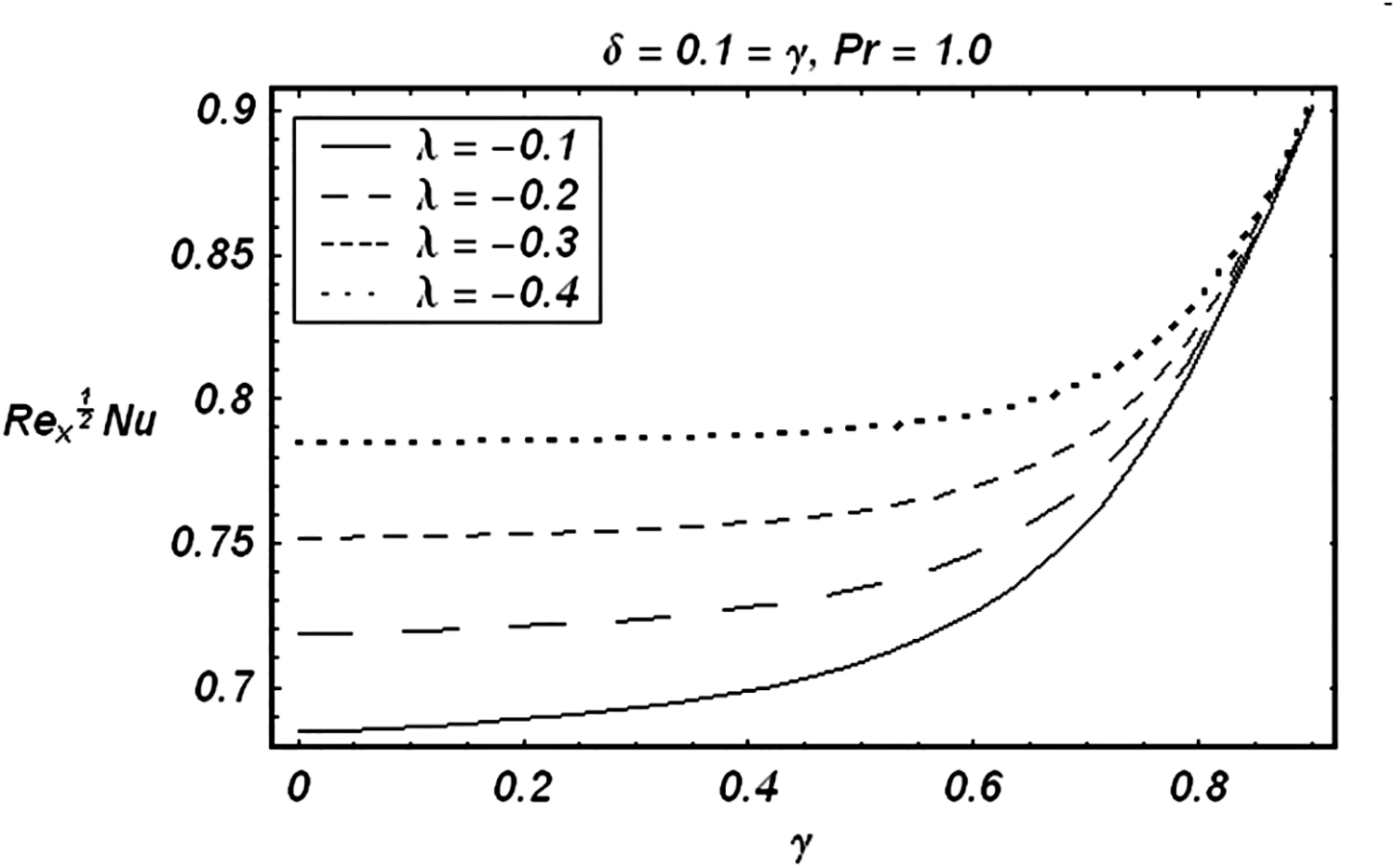
Effect of (λ<0) on local Nusselt number.

Tables 2 and 3 are presented to analyze the comparison of HAM and numerical solutions for various values of embedding parameters for −*f*″(0) and −*θ*′(0) respectively. A comparative study of these two tables shows an excellent agreement. Our interest further is concerned with the influence of parameters *ε*, *δ*, *γ*, *λ* and Pr on the velocity and temperature fields. Hence we draw the Figs 3–12 for such objective. Figs 3 and 4 are plotted to examine the variations of *δ* and *ε* on the velocity field. We see from (Fig 3) that the velocity decreases when *δ* is increased. The influence of parameter *ε* on the velocity is quite opposite to that of *δ* (See Fig 4). Effects of heat source (*λ* > 0) and sink (*λ* < 0) on the temperature are analyzed in the Figs 5 and 6. As expected, (Fig 5) illustrates that there is rise in temperature when *λ* > 0. However, the temperature decreases when *λ* < 0. Effects of Pr on temperature is plotted in (Fig 7). Here the temperature decreases when we increase the Prandtl number Pr. This is because of the reason that an increase in Pr decreases the thermal conductivity of the fluid and consequently the temperature decreases. (Fig 8) displays the effects of conjugate parameter *γ* on temperature *θ*(*η*). It is observed that temperature is an increasing function of. (Fig 9). Show the variation of *δ* on skin friction coefficient when other parameters are kept fixed. It is noticed that skin friction coefficient increases by increasing parameter *δ*. Figs 10, 11 and 12 respectively plot the variation of Pr, (*λ* > 0) and (*λ* < 0) on the local Nusselt number. These Figs. Witness that the local Nusselt number increases by increasing Pr and *λ* < 0. However, the behavior of *λ* > 0 is reverse when compare with Pr and *λ* < 0.

**Table 2:** Comparison of the values of –*f*″(0) by HAM with the numerical solution for various values of *ε* and *δ*.

E	δ	HAM Solution	Numerical Solution	
			bvp4c	Shooting Method
0.1	0.1	0.956018	0.956017	0.955955
0.2		0.917972	0.917970	0.917970
0.3		0.883221	0.883224	0.883225
0.1	0.1	0.956018	0.956017	0.955955
	0.5	0.964859	0.964862	0.964862
	1.0	0.975361	0.975312	0.975310

**Table 3:** Comparison of the values of –*θ*′(0) by HAM with the Numerical solution for various values of *γ*, Pr and *λ*.

Γ	Pr	Λ	HAM Solution	Numerical Solution	
				bvp4c	Shooting Method
0.1	1.0	0.2	0.139597	0.139480	0.138236
0.2			0.434178	0.434174	0.434268
0.3			1.523070	1.523070	1.521310
0.1	1.0	0.2	0.139597	0.139480	0.138236
	2.0		0.117307	0.117301	0.117533
	2.5		0.115094	0.115012	0.114490
0.1	1.0	0.1	0.125670	0.125670	0.125543
		0.2	0.139597	0.139574	0.138150
		0.3	0.203966	0.203966	0.203967
